# A paradigm change to inform fibromyalgia research priorities by engaging patients and health care professionals

**DOI:** 10.1080/24740527.2017.1374820

**Published:** 2017-10-23

**Authors:** Mary-Ann Fitzcharles, Mary Brachaniec, Lynn Cooper, Ruth Dubin, Trudy Flynn, Kerstin Gerhold, Winfried Häuser, Katherine Cowan, Andreas Laupacis, Renee Marleau, Marc Milot, Nicole Szajcz-Keller, Janice Sumpton, Zach Walsh, Hani El-Gabalawy

**Affiliations:** a Division of Rheumatology and Alan Edwards Pain Management Unit, McGill University Health Centre, Montreal, QC, Canada; b Riverview, NB, Canada; c Kitchener, ON, Canada; d Department of Family Medicine, Queen’s University, Kingston, Canada; e Seabright, NS, Canada; f Department of Pediatrics and Child Health, University of Manitoba, Winnipeg, Canada; g Department of Internal Medicine 1, Klinikum Saarbrücken, Saarbrücken, Germany; h Department of Psychosomatic Medicine and Psychotherapy, Technische Universität München, Munich, Germany; i James Lind Alliance, London, England; j Li Ka Shing Knowledge Institute of St. Michael’s Hospital, Toronto and Faculty of Medicine, University of Toronto, Toronto, ON, Canada; k Terrasse-Vaudreuil, QC; l APAS Laboratory, Montréal, QC, Canada; m Canadian Institutes for Health Research, Institute of Musculoskeletal Health and Arthritis, Winnipeg, MB, Canada; n Department of Pharmacy, London Health Sciences Centre, London, ON, Canada; o Department of Psychology, University of British Columbia, Kelowna, BC, Canada

**Keywords:** fibromyalgia, management, shared decision making

## Abstract

**Background**: Research objectives should be focused toward advancing knowledge that has meaningful impact on health. However, research agendas are mostly driven by the health care community, with limited input from patients.

**Aims**: In this study, prioirities of uncertainties for the management of fibromyalgia (FM) that could propel future research were identified by a defined process using the James Lind Alliance Priority Setting Partnership (JLA-PSP) methodology.

**Methods**: As a first step, a survey was distributed across Canada that engaged patients, caregivers, and health care professionals to provide narrative input to eight open-ended questions regarding FM care. Responses were thematically condensed and synthesized into an initial list of 43 uncertainties used to guide a comprehensive literature search. Questions already effectively addressed in the literature were excluded, leaving 25 uncertainties that were ranked during a one-day consensus workshop.

**Results**: Three broad themes emerged: the value of personalized targeted treatment and subgrouping of patients; the efficacy of various self-management strategies and educational initiatives; and identification of the ideal health care setting to provide FM care. Opioids and cannabinoids were the only specific pharmacologic interventions ranked as needing further research.

**Conclusions**: The prioritized questions highlight the importance of recognizing the heterogeneity of FM symptoms, the need for a personalized treatment approach, and a better understanding of the value of self-management strategies. This is the first study that uses an established and transparent methodology to engage all FM stakeholders to help inform researchers and funding bodies of clinically relevant research priorities.

## RÉSUMÉ

**Contexte**: Les objectifs en matière de recherche devraient se concentrer sur l’avancement des connaissances qui ont des effets significatifs sur la santé. Toutefois, les programmes de recherche sont surtout définis par le milieu des soins de santé, tandis que la contribution des patients demeure limitée.

**Objectifs**: Dans cette étude, les incertitudes prioritaires pour la prise en charge de la fibromylagie, qui pourraient donner lieu à de futures études, ont été déterminées selon un processus fondé sur la méthodologie du Partenariat d’établissement des priorités de la James Lind Alliance.

**Méthodes**: Comme première étape, une enquête a été distribuée partout au Canada auprès de patients, de prestataires de soins et de professionnels de la santé afin d’obtenir leurs réponses sous forme narrative à huit questions ouvertes concernant les soins relatifs à la fibromyalgie. Les réponses ont été regroupées par thèmes et résumées dans une liste initiale de 43 incertitudes qui a été utilisée pour orienter une recherche exhaustive de la littérature. Les questions déjà abordées de manière efficace dans la littérature ont été exclues, tandis que les 25 incertitudes restantes ont été classées dans le cadre d’un atelier de recherche de consensus d’une journée.

**Résultats**: Trois grands thèmes se sont démarqués : l’utilité des traitements ciblés personnalisés et de la division des patients en sous-groupes; l’efficacité de diverses stratégies d’auto-prises en charge et initiatives éducatives; et la détermination du cadre de soins de santé idéal pour dispenser les soins relatifs à la fibromyalgie.

**Conclusions**: Les questions priorisées soulignent l’importance de reconnaître l’hétérogénéité des symptômes de la fibromyalgie, la nécessité d’une approche de traitement personnalisé et une meilleure compréhension de l’utilité des stratégies d’auto-prise en charge. Il s’agit de la première étude à utiliser une méthodologie établie et transparente pour impliquer toutes les parties concernées par la fibromyalgie pour aider à faire connaître aux chercheurs et aux agences de financement les priorités pertinentes sur le plan clinique.

## Introduction

Shared decision making between patients and health care professionals is increasingly important in medicine, with patient empowerment contributing to improved clinical care and outcome for many conditions. The patient voice in determining a research agenda is, however, a lesser known but emerging concept. Acknowledging the importance of the patient’s lived persoanl perspective, this evolution of thinking should now extend to patient contribution in setting a research agenda. Substantial input from patient stakeholders in the priority-setting process should ensure that research is clinically meaningful and directly applicable to care. This novel concept is particularly relevant for directing research in fibromyalgia (FM), a condition with many unanswered questions but specifically those surrounding ideal management that may ultimately improve patient outcomes.^1–3^

An innovator in this area of patient engagement has been the James Lind Alliance (JLA), a United Kingdom–based iniative that has developed a standardized methodology to involve various key stakeholders in identifying unanswered questions or “uncertainties” in clinical care that can help direct a research agenda.^4–6^ Partnering with JLA in a research priority-setting partnership (PSP) provides the opportunity to recognize real-life gaps in clinical care and thereby propel research that will improve symptoms and health related quality of life (HRQoL) for various medical conditions. This process is unique in that both quantitative and qualitative information is combined in a standardized process to reach a final objective of ten prioritized questions that remain unanswered by current research.

FM is common worldwide, affecting at least 2% of the population. It is characterized by widespread body pain with associated core symptoms of fatigue, sleep disturbance, and cognitive problems as well as other somatic and mood complaints.^2,7,8^ Clinical criteria and severity scales for FM were defined by the American College of Rheumatology (ACR) in 2011, with further revision in. 2016^9,10^ The exact cause and pathogenesis of FM are mostly unknown.^2,9^ Effective treatments for FM are elusive, with no current “gold standard” of care and an expectation that symptoms will likely persist throughout life. Symptoms of FM have immediate effects on HRQoL and personal and social functioning. FM incurs both direct and indirect health costs.^11–14^ Body pain and fatigue, pivotal symptoms in FM, are also important components of many other medical and especially rheumatic complaints, emphasizing the need to specifically address symptoms in clinical care of patients with FM. Although mostly recognized as a unique diagnosis, FM may also be a comorbid condition associated with other rheumatic, somatic, and mental disorders, thus broadening the impact of this condition.^8,15^

The aim of this adult FM PSP was to engage patients, caregivers, and health care providers to identify the scope of uncertainties relating to the effects of treatments for adult FM patients and to agree on a prioritized list of the top ten uncertainties that can be used by researchers and funding bodies to direct clinically meaningful research to improve the care of persons with FM.

## Methods

Throughout this exercise we followed the JLA-PSP process according to the recommendations set out in *The James Lind Alliance Guidebook*, Version 5,^4,^^5,^^6^ and throughout the process we were guided and advised by a senior advisor for JLA (CK). Following a Canadian workshop hosted by the Canadian Institutes for Health Research, Institute of Musculoskeletal Health and Arthritis (CIHR-IMHA) addressing chronic pain and fatigue, a 12-person steering committee (SC) was created. The steering committee was led by the first author of the *Canadian 2012 Fibromyalgia Guidelines*, ^16^ and members were recruited after consultation with health care professionals and patient groups across Canada, who had either been involved in the guideline development or were members of organizations known to have an interest in FM. The steering committee was composed of five patients with FM (one patient was a practicing pharmacist), five health care professionals experienced in treating persons with FM (one family physician, two rheumatologists, one psychologist, one internist), an internist with previous experience of the JLA process but without specific interest in FM, and the scientific director of IMHA, who is a rheumatologist, and was convened by CIHR-IMHA. A consultant from the JLA participated as an observer to ensure accountability and transparency throughout the process. Four CIHR-IMHA staff provided data analysis and administrative support and the SC met by telephone conferences on 15 occasions over a 16-month period.

### Identifying uncertainties via a national survey

A national online anonymous survey, distributed via partner organizations, societies, and social media, was used to collect research priorities identified by patients, caregivers, and clinicians regarding the management of FM. A total of 109 groups/organizations/bodies were contacted as well as the original 139 voting participants in the *Canadian 2012 Fibromyalgia Guidelines*.^16^ In total, 17 organizations participated (five professional associations, five pain centers, and seven patient groups). The survey, in English and French, included 16 questions, eight demographic and eight open-ended questions requesting a narrative response. The demographic questions included participant type (patient, caregiver, clinician, member of an organization or support group representing people with FM), age, sex, geographic region in Canada, and language (French or English). Patients with FM were invited to participate in the survey if they “had been diagnosed with FM,” although the diagnosis was not further validated. Those with FM reported the duration of chronic widespread pain and time since FM diagnosis, and health care professionals reported professional type and work setting. Open-ended questions were modeled on those previously used in PSP studies and were developed by the SC with a focus on FM. Beginning with a general question about the diagnosis and management of FM, there followed seven more probing themed questions that prompted participants in a nonbiased way. These included questions about health care provision, medications, lifestyle practices that have an impact on specific symptoms, and HRQoL. The survey received the University of Manitoba’s Health Research Ethics Board approval, with completion of the survey taken as implicit consent to participate in the PSP.

Narrative responses were reviewed in detail, and those out of scope were removed (e.g., Is there a genetic predisposition to fibromyalgia? Are there any permanent changes in the brain of a person with fibromyalgia? What is the estimated cost of fibromyalgia to insurance companies and the federal government in disability payments?). Those in scope were grouped according to themes, duplicates were combined, and a series of “indicative questions” that could potentially be addressed by research was developed that reflected the responses to the survey (set 1).

### Evaluation of current knowledge pertaining to questions generated

The James Lind Alliance process excludes from the final workshop questions that are considered “answered” by relevant, reliable, and up-to-date systematic reviews. The following step was to determine to what extent the set 1 questions (*n* = 43) had been answered by previous research. The JLA guidelines recommend consulting Cochrane systematic reviews as well as those from the Database of Abstracts of Reviews of Effects (DARE). All up-to-date Cochrane reviews (including protocols) related to fibromyalgia as well as those from the DARE were collected (December 2015); 119 reviews were identified as relevant to fibromyalgia and acquired. A number of guidelines composed of systematic reviews or meta-analyses related to fibromyalgia were also collected (*n* = 10) and consulted if the question pertained to guidelines. The primary step in the review process consisted of reading the reviews, tagging them (if applicable) to one or more of the 43 research questions they addressed, and then determining whether each question was adequately answered by applicable Cochrane or DARE reviews. An “answered” question was defined as one not requiring future research when the majority of the Cochrane or DARE reviews pertinent to the question indicated no need for future research or did not highlight significant issues surrounding existing research, such as low-quality design or limited clinical applicability. Additional information to determine whether questions were answered was obtained by a review of primary research trends and an online survey of two FMS experts. To further ensure that all relevant information had been fully accessed by the primary review of Cochrane and DARE, a further supplementary analysis of primary research trends was conducted. For this second check process, a search of titles or abstracts published since 2000 was conducted (December 2015) in Scopus, Web of Science, and PsycINFO and a database of 5127 abstracts was built. A random selection of 20% of the abstracts was selected for review (*n* = 1032) to ensure that no additional information was outstanding. The review process consisted of reading each abstract and tagging it (if applicable) to one or more of the 43 research questions it was considered to address. For the “expert” survey, two health care professional members of the SC (MAF, WH), with expertise in FM, completed an online questionnaire (March 2016) and rated the degree of impact of further research on how each of the 43 questions was currently answered by research and provided open-ended feedback and clarification.

An impact score (IS; see Table 1) of future research for each of the 43 research questions was generated based on all sources of information (consultation of Cochrane and DARE reviews/guidelines, primary research trends, and expert survey). The IS quantified the degree to which each research question was considered answered and was based on the estimated likelihood that future research would change or provide further clarification on how a question is currently answered. For the analyses, a low IS was considered to indicate a higher likelihood that a question was currently answered by existing research. A score of 0 was assigned if the consultation of Cochrane and DARE reviews indicated that a research question was adequately answered. For all other questions considered as not answered by this initial consultation of reviews, an overall average IS was calculated based on two separate impact scores generated from the review of the primary research trends and from the expert survey (both scored on the same grading system/scale; Table 1). For the primary research trends, the IS was based on the number of studies addressing a particular question, with questions having a lower number of studies considered as being less likely to be answered by existing studies (higher impact score). The IS based on expert feedback was the average of the two scores provide for each question. A high concordance was observed between the impact scores generated from the expert feedback and those based on the primary research trend analysis. Any questions with a very low overall IS (considered as closer to being answered by existing research) were removed to leave set 2 questions that could be further condensed by the SC to a final set 3 of a short list of 25 questions to be brought forward to a prioritization determining workshop.

**Table 1.** Impact score and interpretation.

Impact score	Score range	Interpretation
3 (High)	3.0	Further research *is very likely to change* how the research question is currently answered; very low probability of currently being answered and closest to being an actual uncertainty
2 (Moderate)	2.0 to 2.9	Further research *is more than likely to change* how the research question is currently answered; low probability of currently being answered
1 (Low)	1.0 to 1.9	Further research *is likely to change* how the research question is currently answered; high probability of currently being answered
0 (very low)	0.0 to 0.9	Further research is not likely to change how the question is currently answered; highest probability of currently being answered and not being an uncertainty

### Research prioirity-setting process

In a one-day in-person workshop, facilitated by a JLA consultant, the 25 questions (set 3) were ranked by consensus. In addition to the SC members, workshop participants were recruited by the SC ensuring equal representation of patients and clinicians, taking into account sex, age, and geographic location. Participants completed a declaration of interest prior to attendance. Persons identified as either patients or clinically active health care professionals were eligible to attend the workshop as per JLA guidelines (nine patients, nine clinicians). There were three workshop facilitators with experience of the JLA process. Prior to the workshop, participants received a randomly ordered list of set 3 (the top 25 questions), which they were asked to rank individually in order to prepare for the task.

Three priority-setting sessions were convened during the workshop day. Each group worked with a set of 25 A4-sized cards onto which the questions were printed. For session 1 and session 2, the 18 participants were assigned to one of three small groups including six participants per group for each session. Each small group included an equal number of patients and clinicians, with differing composition of small groups for session 1 and session 2. All participants were present for the final session 3. For session 1, participants identified their top and bottom three questions of set 3, with information recorded by the facilitators, and, after a short break, questions were organized into three sections: top, middle, and bottom. In each small group, participants were encouraged to share and discuss their views on the questions and to agree by consensus the ranked order of all 25. For session 2, each small group focused on the 25 questions as ranked after combining the results of session 1. Again, through a process of discussion and consensus decision making, the 25 questions were ranked. A session 2 overall ranking was calculated by combining the results of all three groups. For session 3 (the final priority-setting session), the participants worked with 25 cards laid out in the combined ranked order determined by session 2, and all participants contributed to a whole-group discussion to agree on the final order of the 25 questions, with emphasis on the top 10 questions. Discussion was invited regarding combining questions and/or modifying wording. Agreement was then reached for a final order of the questions, highlighting the top ten research priorities in the management of FM in adults.

## Results

Five hundred and fifty online surveys were returned over a 12-week period between July and October 2015. Becaus the survey was posted on the websites of various partner organizations and individuals were not specifically targeted, except for the voting members of the *2012 Canadian Fibromyalgia Guidelines*,^16^ a response rate to the survey cannot be calculated. The demographic information for participants is shown in Table 2. Responses were returned from all provinces across Canada, with about two thirds completed in English and two thirds completed by women. Over 80% of patients had experienced widespread body pain for at least 5 years, with two thirds reporting a diagnosis of FM for at least 5 years. Demographic information was lacking or the questionnaire was incomplete in about one quarter of questionaires.

**Table 2.** Profile of survey respondents.^a^

	Overall	Patients	Clinicians
Variable	(*n* = 550)	(*n* = 292)	(*n* = 109)
Type of participant, *n* (%)			
Patient	292 (53)		
Caregiver	14 (3)		
Clinician	109 (20)		
Organization member	13 (2)		
Not identified	122 (22)		
Age, *n* (%)			
18–39 years	60 (11)	37 (11)	20 (18)
40–49 years	85 (15)	56 (19)	23 (21)
50–59 years	148 (27)	111 (38)	28 (26)
60–69 years	91 (17)	63 (22)	23 (21)
70 years or older	38 (7)	21 (7)	11 (10)
Not identified	128 (23)	4 (1)	4 (4)
Sex, *n* (%)			
Male	62 (11)	13 (4)	44 (40)
Female	351 (64)	273 (93)	54 (50)
Not identified	137 (25)	6 (2)	11 (10)
Region, *n* (%)			
Atlantic	34 (6)	25 (9)	8 (7)
Quebec	182 (33)	141 (48)	28 (26)
Ontario	90 (16)	46 (16)	41 (38)
Prairies	34 (6)	17 (6)	13 (12)
British Columbia	79 (14)	58 (20)	14 (13)
Territories	1 (<1)	1 (<1)	0
Not identified	130 (24)	4 (1)	5 (5)
Language, *n* (%)			
English	336 (61)		
French	214 (39)		
Health care professional, *n* (%)			
Rheumatologist			49 (45)
Family physician/general practitioner			13 (12)
Physiotherapist			9 (8)
Psychologist			7 (6)
Social worker			4 (4)
Psychiatrist			4 (4)
Anesthesiologist			4 (4)
Chronic pain specialist			5 (5)
Other			11 (10)
Not identified			3 (3)
In which setting do you primarily work?			
Academic			45 (41)
Clinical			47 (43)
Community			14 (13)
Not identified			3 (3)

^a^Values presented in the table are the number of respondents; proportions are included in the brackets. Demographic questions were asked based on the type of respondents; not all respondents answered all demographic questions.

**Figure 1.**
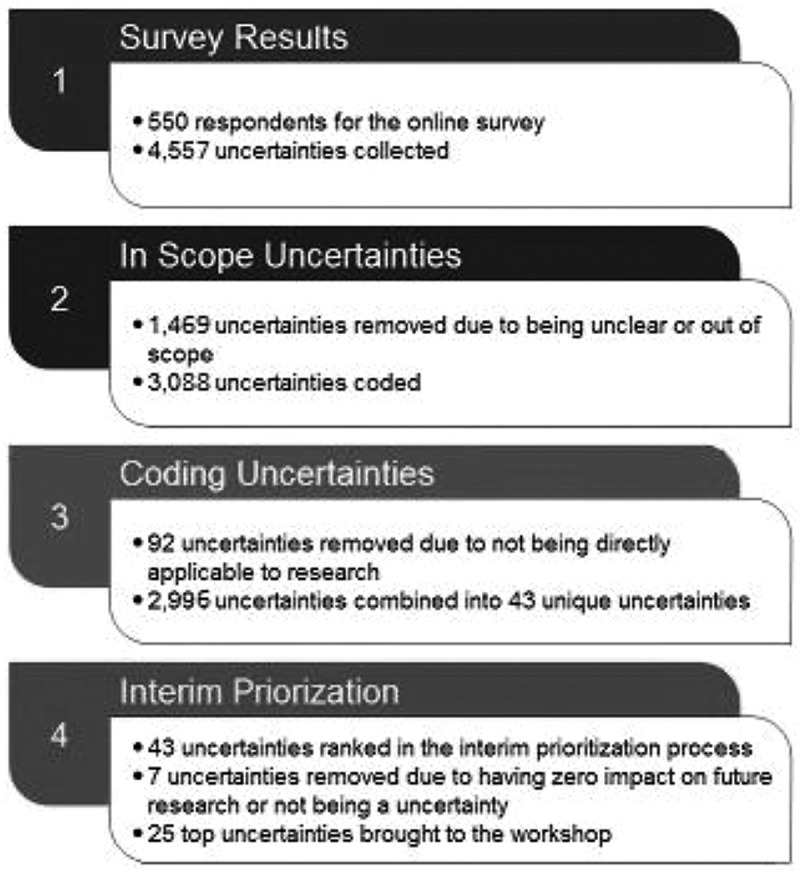
Process of combining and prioritizing management uncertainties for fibromyalgia.

The process of combining and prioritizing management uncertainties is shown in Figure 1. There were 4557 lines of written commentary identifying uncertainties that included questions, comments, and stories. Each line of narrative was read by two research assistants, commonly themed topics and questions were grouped, duplicates were aggregated, and narrative that was not in the form of a question was examined to determine whether an embedded question could be recognized. The frequency of in-scope uncertainties were grouped according to themes (Table 3), with one third addressing pharmacologic and complementary treatments, one third lifestyle factors and education, 10% health system services, and 3% work and employment. Themes and questions that did not address management were set aside. Fifty-five uncertainties with accompanying reference narrative were formulated and examined for clarity and repetition by two members of the SC with expertise in FM (MAF, WH) and further condensed to 43 uncertainties (set 1).

**Table 3.** Frequency of in-scope uncertainties, by theme.^a^

Variable	All narratives (*n* = 2996)
Pharmacologic treatments/medication	588 (20)
Alternative/complementary treatments	506 (17)
Lifestyle	484 (16)
Education	444 (15)
Guidelines/best treatments	349 (12)
Health systems services	335 (11)
Cognitive/psychological	175 (6)
Work/employment	90 (3)
Personalized medicine	78 (3)
Hypersensitivity/allergies	37 (1)
Other factors (sex, physical environment)	35 (1)
Other	40 (1)

^a^Respondents could provide more than one response; totals may sum to more than 100%. Values presented in the table are the number of narratives; proportions are included in brackets.

The 43 questions (set 1) were used to explore the current literature to determine whether they remained an uncertainty. As a first step for the literature review, all 119 relevant reviews (Cochrane and Dare) and ten clinical guidelines were identified, full articles were read, additional references arising from the reviews were identified, and 348 articles were tagged to a research question. As an additional literature check, a search of titles or abstracts published in Scopus, Web of Science, and PsycINFO yielded 5127 abstracts, of which a random selection of 1032 articles (20% of total) were scanned (titles/abstracts), and articles were tagged to a question where appropriate, with 279 instances of tagging.

Each question of set 1 was graded for degree of uncertainty according to the literature review and the expert review, “published” IS and “expert” IS, respectively, and a “combined” IS was calculated for each question. A high concordance was observed between the published IS and the expert IS. Notably, 80% of the top 25 questions (ranked by highest IS) identified by the published IS were also in the top 25 questions based on the experts’ average score (expert IS). The combined IS for seven questions in set 1 was very low (a score of 0.0 to 0.9) and they were therefore removed, leaving a total of 36 questions (set 2). To develop a short list of 25 questions (set 3) for final priority setting, an interim ranking of the top 15 questions in set 2 was done by the SC (five patients and five clinicians), with a final interim rank score assigned to each question. These 25 questions were brought forward to the prioritizing workshop.

The top ten identified priorities for research for the management of FM are shown in Table 4, with examples of narrative that identified the question, as well as the relative contributions of health care professionals and patients for each question. At the final workshop, three of the 25 questions were combined into a single question, which was rated as priority 1, “Can early targeted/personalized treatment plans based on subgrouping and/or staging of severity improve outcome for people living with fibromyalgia?” Self-management strategies emerged as four priorities, including identification of optimal exercise activity and/or dietary recommendations, education of patients to actively participate in care, innovative self-management tools including use of social media, and the value of improved health literacy, (priorities 2, 4, 6, and 9, respectively). Three specific symptoms requiring further study for treatment recommendations were sleep disturbance, cognitive effects, and hypervigilance (priorities 3, 7, and 10, respectively). Research to identify the optimal health care setting as well as care trajectory in continued care was rated as priority 5. The only specific pharmacologic interventions that were prioritized as requiring further study was the need to evaluate both safety and efficacy of opioids and cannabinoids in the treatment of FM.

**Table 4.** Top ten research uncertainties.^a^

	Research uncertainty	Type of participants
1	Can early targeted/personalized treatment plans based on subgrouping and/or staging of severity improve outcome for people living with fibromyalgia?	Source of uncertainty: A similar proportion of patients and clinicians identified this uncertainty.
		Sample narrative:
		Any given sufferer will not have all of the possible symptoms but would some tend to be seen together more often than others? Could this lead to identification of a number of subsets? This way, if a physician determines one symptom, she or he could more quickly ask about others in the subset. This in turn might open up possibilities regarding more targeted treatments of symptoms within subsets.
		Why one medication works better than others for specific people?
		Would like to see the ability to predict who will benefit most from a given medication without having to take it first?
		Combined uncertainties:
		Can targeted/personalized treatment plans improve outcomes for people living with fibromyalgia?
		Would subgrouping and/or staging fibromyalgia patients define specific therapies and improve patient health outcomes?
2	What evidence is there to support the use of lifestyle interventions (i.e., nutrition, exercise, take more breaks, general lifestyle interventions) for the management of fibromyalgia symptoms?	Source of uncertainty: A higher proportion of patients compared to clinicians indicated this uncertainty.
		Sample narrative:
		What types, frequency, and intensity of exercise is best for people with fibromyalgia?
		Are there dietary recommendations that have proven effective as treatment options?
		Do those who modify their lifestyle experience relief from their fibromyalgia?
3	What are the best ways to manage sleep problems in people living with fibromyalgia?	Source of uncertainty: A higher proportion of clinicians compared to patients stated this uncertainty.
		Sample narrative:
		How is sleep deprivation key to creating/influencing fibromyalgia symptoms?
		What is the best/most successful treatment to improve sleep quality?
		Are antidepressants as effective as or more effective than other medications used as a sleep aid?
4	What are the effective methods for educating patients living with fibromyalgia to take an active role in their care?	Source of uncertainty: A similar proportion of patients and clinicians reported this uncertainty.
		Sample narrative:
		What kind of information is available to the newly diagnosed fibromyalgia patient?
		How can physicians and health care providers better communicate about fibromyalgia to patients and caregivers?
		What communication skills are helpful in convincing fibromyalgia patients to start exercising?
5	What are the health care settings for persons with fibromyalgia that would allow for the best health care professional and optimal care pathway and for appropriate follow-up?	Source of uncertainty: A higher proportion of clinicians compared to patients identified this uncertainty.
		Sample narrative:
		Who will be the most qualified medical personnel to help a person diagnosed with fibromyalgia?
		How can we get the proper psychosocial support for the biomedical model of pain for these patients?
		How access to quality health care impacts the quality of life of someone who suffers with fibromyalgia?
6	What innovative self-management strategies, including social media and online tools, may be used in fibromyalgia care and do they impact outcome?	Source of uncertainty: A slightly higher proportion of clinicians compared to patients stated this uncertainty.
		Sample narrative:
		How can we implement effective self-management programs using novel technologies (e.g., social media)?
		Would chronic pain and activity type videos such as “a really good video on chronic pain” as found on rheumatology info website or those in the style of Michael Evans from the University of Toronto have effect?
		Can research help identify and make more accessible resources for those who are mostly housebound and isolated?
7	What are the best methods to treat and manage cognitive symptoms of fibromyalgia?	Source of uncertainty: A slightly higher proportion of patients compared to clinicians indicated this uncertainty.
		Sample narrative:
		Are there things available (besides rest or sleep) to alleviate “fibro fog” or failing that are there things to do to decrease the patients susceptibility to this fibro fog?
		Are there effective pharmacological treatments for memory difficulties?
		What kind of treatments/exercises/routines can be done to help improve concentration?
8	How safe and effective is the use of cannabinoids and opioids in treating fibromyalgia?	Source of uncertainty: A higher proportion of clinicians compared to patients identified this uncertainty.
		Sample narrative:
		Will research help determine whether opiate medications are actually effective in treating pain?
		What are the long-term effects of cannabinoid therapy on people with fibromyalgia?
		Does smoking marijuana affect sleep, pain, or other symptoms of fibromyalgia?
9	Does improving patient health literacy (i.e., education on medications, neuroscience of pain mechanism) help improve patient health outcomes in people with fibromyalgia?	Source of uncertainty: A similar proportion of patients and clinicians stated this uncertainty.
		Sample narrative:
		What is the effect of neuroscience pain education (delivered by physiotherapists) on perceived functional abilities?
		What different types of medications are out there for treatment (as I’ve only been offered antidepressants)?
		How effective is a “sit down” course like Chronic Pain Self-Management or Chronic Disease Self-Management as offered by the University of Victoria in British Columbia in the long term?
10	What is the most effective treatment for hypersensitivity (e.g., touch, noise, odor, light, hypervigilance) in fibromyalgia patients?	Source of uncertainty: A similar proportion of patients and clinicians indicated this uncertainty.
		Sample narrative:
		Why people with fibromyalgia have more chemical sensitivities or are more prone to side effects of medications?
		How to reduce the fight or flight response?
		How should the incredible skin sensitivity of fibromyalgia to any light touch or pressure be treated/alleviated?

^a^Source of uncertainty was reported only on patients and clinician responses because the *n*-size for caregivers and patient/organization groups was small.

## Discussion

This is the first study with equal voice of patients, caregivers, and health care professionals that provides a proposed set of clinically relevant questions that may direct a research agenda for the management of FM. This exercise identified three broad domains pertaining to the treatment of FM that had to date not been sufficiently answered, required further research, and were most likely to meaningfully impact the HRQoLof persons with FM. The domains were (1) a need to examine the value of an early personalized treatment plan with targeting of specific symptoms, (2) a better understanding of various self-management strategies, and (3) identification of the ideal health care setting for patients. The only specific drug intervention identified as an uncertainty of high priority was the use of opioids and cannabinoids. This study highlights the personal impact of a condition, characterized by heterogeneous complaints not yet sufficiently addressed; a need and willingness for patients to be proactive in health care by exploring self-management and educational strategies; and the quandary of responsibility for best clinical care. Specific drug treatments, other than opioids and cannabinoids, were not rated as areas of high priority requiring additional research. Although the focus of this exercise was to identify the top ten questions, we have considered all 23 questions that were analyzed at the consensus workshop.

The first theme was the need to address individualized treatment approaches, taking into account personal characteristics and condition severity. Recognizing the heterogeneity of symptoms experienced by individuals should inform clinical care, particularly because the traditional focus of FM management has mostly addressed pain, with lesser attention to other symptoms. This is particularly true of pharmacotherapeutic studies, especially in the categories of antidepressants and gabapentinoids. The need to examine personalized treatments emphasizes the variability in symptoms of FM, with individual symptoms weighted differently for different persons. Sleep disturbance, cognitive symptoms, and symptoms of hypersensitivity or hypervigilance to various stimuli were specifically identified within the top ten priorities requiring further study. This therefore raises the question of treat-to-target and focus on symptoms that meaningfully impact well-being of FM patients, a concept that has only recently entered the dialogue of FM care.^3^

The second theme was to examine self-management strategies such as lifestyle modifications, educational techniques including use of social media, and methods to improve health literacy. Social media is increasingly used by patients but with valid concerns about the reliability of information accessed. In this age where increasing numbers of patients are technologically savvy, the use of social media as an educational or treatment tool must be further examined. These questions speak to the need for active patient participation in health practices as has been strongly recommended by recent guidelines for the management of FM.^16–19^

Whether persons with FM should ideally be managed by primary care physicians, specialists, or multidisciplinary teams is not known. This uncertainty was reflected by allocation as priority 5 in this exercise. This issue has challenged patients and health care providers alike, with a prevailing belief in North America that medical care is better provided in a specialist care setting, rather than by family physicians. Similarly, treatments administered by a multidisciplinary team may be reassuring for patients but is neither accessible for all nor has been shown to be superior to care provided by competent and educated primary care physicians. High prevalence of FM as well as burdening concerns of rising health care costs require futher study of the effects of multidisciplinary care.

The only specific drugs identified as needing further research were the categories of opioids and cannabinoids. These drug categories are important for a number of reasons. Opioids have become increasingly prominent over the last 2 decades for the management of painful conditions but with increasing concern about the risks associated with long-term opioid therapy, including addiction and overdose. In contrast, cannabinoids are emerging as a potential substitute for opioids in the treatment of pain-related conditions.^20^ In this context, neither of these drug categories has ever been effectively examined in the management of FM symptoms. Unfortunately, drug treatments for symptom management for FM have failed to fulfill earlier hopes, with current drug treatments recognized as imperfect, offering mostly modest benefit for the majority of patients, with only a few experiencing substantial effect.^2^ A recent network meta-analysis of all treatments for FM reported that the average benefits of pharmacologic treatments were of questionable clinical relevance and that the evidence for nonpharmacological interventions is limited.^21^

The majority of questions in set 1 were considered unanswered by the initial review of Cochrare/DARE review and use of the combined IS confirmed that most questions were closer to being uncertainties, with only a single question identified in this process considered as answered. This assessment was corroborated by concordance between the formal literature search and expert input. Recent evidence-based guidelines for the management of FM published for Canada, Germany, Israel, and the European League Against Rheumatism attest to the relative lack of sound evidence on which to base some recommendations.^19,22^ It is therefore hoped that the uncertainties raised by this current JLA endeavor will add further clarification for treatment options for FM and that the current knowledge gap will be addressed.

There are a number of limitations to this study that are acknowledged. Firstly, the diagnosis of FM was by self-report and was not validated by face-to-face encounter or review of medical records. Although participants were eligible if they had been diagnosed with FM, this condition may be overdiagnosed or even misdiagnosed as FM when some other medical condition is the cause of chronic widespread pain. Secondly, the response rate by both patients and clinicians across the provinces of Canada was uneven. Although Quebec comprises about one quarter of the population of Canada, the total response rate from Quebec was one third of all responses, with almost half of the patient responses arising from that province. We received responses from all regions of Canada but acknowledge that the province of Quebec was overrepresented, with one third of responses arising from this province. However, the geographic distribution of health care providers who responded to the survey is more in line with the population distribution, with a 26% response from Quebec and a 38% response from Ontario (the two largest Canadian provinces). Therefore, in view of the uneven geographic response rate from the patient group in particular, our results may not be entirely representative of the opinions of all Canadians with FM. It is also notable that a quarter of respondents did not identify their region in Canada. Persons not associated with a specific professional or patient organization would not have been prompted about the survey unless they spontaneously accessed one of the open websites. Although FM is a condition seen mostly in primary care in Canada, there was limited response from primary care physicians, with considerable representation from rheumatologists. It is also not known whether priorities may be different in other countries with other cultures and health care systems. Because responses were in narrative form, a large amount of narrative data was obtained that required personal interpretation into themes, with the possibility of misinterpretation. Because this initiative was developed to solely identify uncertainties in management of FM, questions regarding pathogenesis, methods of diagnosis, and long-term outcome were not addressed. Despite JLA having a standard methodology, the depth of process was at times lacking, such as no clearly defined methodology for determining whether a question is answered or not. It is for this reason that a set of analyses was developed for the current study with the aim to provide a quantitative (or at least semiquantitive) approach to grading an uncertainty, a process that also had its limitations.

By adhering to the predefined JLA process, we have identified unanswered questions about treatments for FM that require research with the aim to improve symptoms and HRQoL of patients. The strength of the JLA strategy is that conclusions are reached by a fair and transparent process, respecting values and integrity, and with equal partnership of patients, caregivers, and health care professionals. We believe that the conclusions of this study are valid in view of the large amount of information obtained from participants drawn from a wide range of domains. There remain important gaps in clinical care from a number of perspectives that include educational needs, strategies for self-management, treatment of specific symptoms, and identification of an ideal health care setting. We hope that the ultimate aim of this study can be achieved by prompting researchers and funders to study questions of management that are clinically relevant for FM patients.
